# A validated heart-specific model for splice-disrupting variants in childhood heart disease

**DOI:** 10.1186/s13073-024-01383-8

**Published:** 2024-10-15

**Authors:** Robert Lesurf, Jeroen Breckpot, Jade Bouwmeester, Nour Hanafi, Anjali Jain, Yijing Liang, Tanya Papaz, Jane Lougheed, Tapas Mondal, Mahmoud Alsalehi, Luis Altamirano-Diaz, Erwin Oechslin, Enrique Audain, Gregor Dombrowsky, Alex V. Postma, Odilia I. Woudstra, Berto J. Bouma, Marc-Phillip Hitz, Connie R. Bezzina, Gillian M. Blue, David S. Winlaw, Seema Mital

**Affiliations:** 1 Genetics and Genome Biology Program, The Hospital for Sick Children, Toronto, ON Canada; 2 Center for Human Genetics, University Hospitals Leuven, Leuven, Belgium; 3 The Centre for Computational Medicine, The Hospital for Sick Children, Toronto, ON Canada; 4 Division of Cardiology, Children’s Hospital of Eastern Ontario, Ottawa, ON Canada; 5 Division of Cardiology, Department of Pediatrics, McMaster Children’s Hospital, Hamilton, ON Canada; 6 Division of Cardiology, Department of Pediatrics, Kingston Health Sciences Centre, Kingston, ON Canada; 7 Division of Cardiology, Department of Pediatrics, London Health Sciences Centre, London, ON Canada; 8 Division of Cardiology, Department of Medicine, Toronto Adult Congenital Heart Disease Program at Peter Munk Cardiac Centre, University Health Network, and University of Toronto, Toronto, ON Canada; 9 Institute of Medical Genetics, University Medicine Oldenburg, Carl von Ossietzky University, Oldenburg, Germany; 10 Department of Congenital Heart Disease and Pediatric Cardiology, University Hospital of Schleswig-Holstein, Kiel, Germany; 11 German Center for Cardiovascular Research (DZHK), Kiel, Germany; 12 Department of Medical Biology, Amsterdam University Medical Center, Amsterdam, The Netherlands; 13 Department of Human Genetics, Amsterdam University Medical Center, Amsterdam, The Netherlands; 14 Department of Internal Medicine, Amsterdam University Medical Center, Amsterdam, The Netherlands; 15 Department of Cardiology, Amsterdam University Medical Center, Amsterdam, The Netherlands; 16 Department of Clinical and Experimental Cardiology, Amsterdam University Medical Center, Amsterdam, The Netherlands; 17 Heart Centre for Children, The Children’s Hospital at Westmead, Sydney, NSW Australia; 18 Sydney Medical School, The University of Sydney, Sydney, NSW Australia; 19 Heart Center, Ann and Robert H. Lurie Children’s Hospital of Chicago and Feinberg School of Medicine, Northwestern University, Evanston, IL USA; 20 Ted Rogers Centre for Heart Research, Toronto, ON Canada; 21 Division of Cardiology, Department of Pediatrics, The Hospital for Sick Children, University of Toronto, Toronto, ON Canada

**Keywords:** Congenital Heart Disease, Genomics, RNA splicing, Non-canonical, Machine Learning

## Abstract

**Background:**

Congenital heart disease (CHD) is the most common congenital anomaly. Almost 90% of isolated cases have an unexplained genetic etiology after clinical testing. Non-canonical splice variants that disrupt mRNA splicing through the loss or creation of exon boundaries are not routinely captured and/or evaluated by standard clinical genetic tests. Recent computational algorithms such as SpliceAI have shown an ability to predict such variants, but are not specific to cardiac-expressed genes and transcriptional isoforms.

**Methods:**

We used genome sequencing (GS) (*n* = 1101 CHD probands) and myocardial RNA-Sequencing (RNA-Seq) (*n* = 154 CHD and *n* = 43 cardiomyopathy probands) to identify and validate splice disrupting variants, and to develop a heart-specific model for canonical and non-canonical splice variants that can be applied to patients with CHD and cardiomyopathy. Two thousand five hundred seventy GS samples from the Medical Genome Reference Bank were analyzed as healthy controls.

**Results:**

Of 8583 rare DNA splice-disrupting variants initially identified using SpliceAI, 100 were associated with altered splice junctions in the corresponding patient myocardium affecting 95 genes. Using strength of myocardial gene expression and genome-wide DNA variant features that were confirmed to affect splicing in myocardial RNA, we trained a machine learning model for predicting cardiac-specific splice-disrupting variants (AUC 0.86 on internal validation). In a validation set of 48 CHD probands, the cardiac-specific model outperformed a SpliceAI model alone (AUC 0.94 vs 0.67 respectively). Application of this model to an additional 947 CHD probands with only GS data identified 1% patients with canonical and 11% patients with non-canonical splice-disrupting variants in CHD genes. Forty-nine percent of predicted splice-disrupting variants were intronic and > 10 bp from existing splice junctions. The burden of high-confidence splice-disrupting variants in CHD genes was 1.28-fold higher in CHD cases compared with healthy controls.

**Conclusions:**

A new cardiac-specific in silico model was developed using complementary GS and RNA-Seq data that improved genetic yield by identifying a significant burden of non-canonical splice variants associated with CHD that would not be detectable through panel or exome sequencing.

**Supplementary Information:**

The online version contains supplementary material available at 10.1186/s13073-024-01383-8.

## Background

Congenital heart disease (CHD) is the most common congenital anomaly, occurring in ~ 1% of live births [1]. Although there is a strong familial and genetic contribution to CHD [2], ~ 90% of sporadic cases with isolated CHD have an unexplained genetic etiology upon conventional clinical testing that is typically limited to exons of known disease-associated genes [3–5]. This suggests that additional mechanisms and variant types may be contributing to the disease [6, 7].

Normal gene function can be disrupted through a variety of mechanisms, including missense variants, premature stop codons, insertions, deletions, or altered RNA splicing. Splice-disruptions may include the loss of wild-type splice junctions and/or the gain of “cryptic” splice sites that create novel exon boundaries, ultimately resulting in disruptions to the normal pattern of RNA splicing which in turn lead to abnormal protein isoforms. Splice-disrupting variants can occur near existing canonical splice sites, in exons, or in deeply intronic regions.

While canonical splice site variants can be identified using conventional sequencing workflows, splice-disrupting variants outside of these sites are more difficult to identify with high confidence. Such non-canonical splice-disrupting variants are reportedly pathogenic in up to 15% of patients with rare genetic disorders [8] but cannot routinely be evaluated by conventional genetic testing. Recent reports have identified non-canonical splice-disrupting variants in CHD and other rare diseases primarily using in silico predictions in exome and genome sequencing data, followed by in vitro validation of their effect using minigene assays [9–11]. However, exome sequencing is unable to detect deeply intronic splice-disrupting variants, and minigene assays alone have technical limitations as a patient-relevant functional assay. Further, current models are not specifically designed to identify cardiac-specific splice-disrupting variants expressed in the human heart. The use of patient myocardium to identify and validate aberrant splicing events has a strong potential to address this gap [12]. Recent American College of Medical Genetics and Genomics and the Association for Molecular Pathology (ACMG/AMP) framework emphasizes that the effect of splice-disrupting variants can be more accurately validated in patient-derived tissue samples [13].

Here we used genome sequencing (GS) and myocardial RNA-sequencing (RNA-Seq) to identify and validate cardiac-specific splice disrupting variants and to develop a heart-specific model for canonical and non-canonical splice variants, which can be applied to patients with CHD. These included patients with two of the most common forms of cyanotic CHD, i.e., tetralogy of Fallot (TOF) and dextro-transposition of the great arteries (TGA). In addition to identifying canonical splice-disrupting variants in known CHD-related genes in 1% cases, this approach identified putatively damaging non-canonical splice-disrupting variants in 11% of isolated CHD, with deeply intronic variants representing 53% of non-canonical splice-disrupting variants in CHD genes. GS was critical for the identification of variants that would not be captured by routine clinical genetic tests including exome sequencing [14, 15], while cardiac RNA-Seq allowed for high specificity in the interpretation of splice-disrupting effects in the heart. This splice-disrupting variant discovery framework, coupled with a case–control burden analysis, provides a practical strategy for increasing the yield of pathogenic splice-disrupting variants in known CHD genes.

## Methods

### Study cohorts

#### Congenital heart disease (CHD) cases

The overall cohort included 1101 probands, of which 875 had tetralogy of Fallot (TOF) and 226 had dextro-transposition of the great arteries (TGA) (Table S1) [16]. Among these cases, 505 TOF and 226 TGA were enrolled through the Heart Centre Biobank Registry at the Hospital for Sick Children (Ontario, Canada), 245 TOF were enrolled through the Kids Heart BioBank at the Heart Centre for Children, The Children’s Hospital at Westmead (Sydney, Australia), and 125 TOF were enrolled through the CONCOR registry at the Amsterdam Medical Center (Netherlands).

CHD patients (*n* = 154) with both DNA for GS and myocardium for RNA-sequencing (RNA-Seq) were divided into a training set for model development, i.e., *Discovery cohort* (*n* = 106) and a set for testing, i.e., *Validation* cohort (*n* = 48). A second validation cohort included 43 unrelated cardiomyopathy probands from Ontario [6]. The *Extension* cohort for model application included 947 unrelated TOF (*n* = 721) and TGA (*n* = 226) probands with GS but without myocardium available for RNA-Seq. Two hundred thirty two family members with GS were additionally used for variant segregation analysis.

Collection and use of biospecimens through the registries was approved by local or central Research Ethics Boards and written informed consent was obtained from all patients and/or their parents/legal guardians and study protocols adhered to the Declaration of Helsinki.

#### Controls

The control cohort included 2570 genome sequencing (GS) data from the Medical Genome Reference Bank (MGRB) [17]. MGRB variants were obtained from the original publication, after alignment to GRCh37 and variant calling for all samples. *Control* cohort characteristics are provided in Table S1.

### Genome sequencing processing, alignment, and variant calling

GS of CHD and cardiomyopathy cases was performed on high-quality DNA from blood or saliva of probands and their family members using the Illumina HiSeq X or NovaSeq platform by The Centre for Applied Genomics (TCAG, The Hospital for Sick Children, Toronto), or Macrogen (South Korea). Illumina TruSeq DNA PCR-Free kits were used for library preparation. GS samples from CHD probands were sequenced to a median average depth of 30.9X (range 12.9–46.6X). In order to identify putatively pathogenic and other DNA variants of interest, GS samples were further processed as follows:

For all *Discovery* cohort, *Extension* cohort, and cardiomyopathy *Validation* cohort GS samples, paired-end raw reads were trimmed and cleaned by trimmomatic v.0.32 [18], then mapped to human reference genome hg38 using bwa v.0.7.15 [19]. The reference genome sequence and training datasets were downloaded from the Genome Analysis Toolkit (GATK) resource bundle (ftp://ftp.broadinstitute.org/bundle) [20]. Mapped reads were realigned and calibrated by base quality score recalibration tools (GATK v4.1.2.0). HaplotypeCaller was used to generate genotype Variant Call Format (gVCF) files for each sample, then gVCF files for batches of samples were combined and joint-called by using CombineGVCFs and GenotypeGVCFs tools. In order to filter out probable artifacts in the calls, single-nucleotide variants (SNVs) and insertion-deletions (indels) were recalibrated separately by variant quality score recalibration (VQSR) tools, and variants that passed VQSR truth sensitivity level 99.5 for SNPs and level 99.0 for indels were retained. The VariantFiltration tool was used to mark out the low Genotype Quality (GQ) SNV and indel sites whose GQ values were lower than 20 and read depths were lower than 10. Copy number variants (CNVs) were called as described in [21], using ERDS [22] and CNVnator [23]. Structural variants (SVs) were called using Manta [24] and Delly [25]. Sample ancestry and relatedness among family members was estimated and verified using somalier v0.2.11 [26] with default parameters.

All CHD *Validation* cohort GS samples were processed using DRAGEN Bio-IT Platform v3.8.4 [27]. Paired-end reads were aligned to hg38 human genome reference (hg38-alt-aware-graph). Small variants (SNV and Indel), CNVs, and SVs were called according to the above DRAGEN workflow. Files in standard output format were generated, with crams for alignment and vcfs for small variants, CNVs, and SVs. Small variant calls were annotated using an Annovar-based workflow and the CNVs and SVs were annotated using custom scripts. Sample ancestry and relatedness among family members was estimated and verified using somalier v0.2.11 [26] with default parameters.

GS data for the MGRB control cohort were generated as previously reported [17]. Briefly, DNA was extracted from blood and Illumina TruSeq Nano DNA High Throughput kits were used for library preparation. Reads were sequenced on Illumina HiSeq X, then aligned to the 1000 Genomes Phase 3 decoyed version of build 37 of the human genome (GRCh37) using GATK best practices. GATK HaplotypeCaller was used to generate gVCFs for SNVs and indels, then joint-called in a single batch using GATK GenotypeGVCFs. All SNVs and indels were converted to hg38 using LiftoverVcf [28].

### Identification and interpretation of pathogenic protein-coding variants

#### Variant identification

To identify putatively disease-causing variants in cases, we first searched for SNVs, indels, and CNVs that were classified as pathogenic or likely pathogenic according to the ACMG/AMP criteria [29, 30]. SNVs and indels were first annotated for pathogenicity using InterVar v2.0.2 [31]. Variants with internal Human Gene Mutation Database (HGMD) Pro 2019 [32] classifications of Disease-associated polymorphism with supporting functional evidence (DFP) were assigned a PS3 score, while variants with an internal classification of disease-associated polymorphism (DP) or disease causing mutation (DM) were assigned a PP5 score. Variants in CHD patients were then mapped to CHD genes.

#### Variant mapping to genes

*CHD gene list*: Tier 1 CHD genes were selected based on a moderate, strong, or definitive association with CHD according to ClinGen criteria[33]. We further annotated and categorized additional CHD genes using (i) published literature; (ii) existing databases including Online Mendelian Inheritance in Man (OMIM) [34], Clinical Genome Resource (ClinGen) [35], and CHDgene [36]; (iii) inclusion in clinical gene panels; and (iv) expert curation. Genes with a limited evidence for association with CHD were classified as Tier 2 genes. This yielded 99 Tier 1 CHD genes with moderate, strong, or definitive associations with CHD according to ClinGen criteria (17 isolated CHD genes, 82 syndromic CHD genes), and 626 Tier 2 CHD genes with limited association with CHD. Canonical transcriptional isoforms were annotated using Matched Annotation from NCBI and EMBL-EBI (MANE) [37]. Gene constraint annotations were obtained from the Genome Aggregation Database (gnomAD) (v2) [38] (Table S2). *Cardiomyopathy gene list*: Variants in cardiomyopathy patients in the validation cohort were mapped to Tier 1 and 2 cardiomyopathy genes that were annotated and classified using ClinGen criteria [35] as previously reported by us [6].

#### SNV and indel classification for pathogenicity

SNVs and indels classified by InterVar as “Pathogenic” or “Likely pathogenic” and occurring in Tier 1 or Tier 2 genes were subsequently manually reviewed. Gene inheritance and associated disease conditions were obtained from OMIM [34]. Variants in recessive genes were required to either have a homozygous or bi-allelic genotype. When full trio GS was available, parental data were used to determine phasing. Otherwise, when variants were in close proximity, DNA reads were searched to determine if variants occurred on different alleles. Ratios of observed/expected (o/e) loss-of-function (LoF) or missense variants for affected genes were obtained from the Genome Aggregation Database (gnomAD) v2.1.1 [38]. Population variant allele frequencies were obtained from gnomAD v3.1.2. Where possible, variant segregation among family members was considered. Variants in genes for dominant disorders had allele frequencies < 0.01% for PM2, between 1 and 5% for BS1, and > 5% for BA1. Variants in genes for recessive disorders had allele frequencies < 0.1% for PM2, between 2 and 10% for BS1, > 10% for BA1. The UCSC Genome Browser was used to investigate low mappability and RepeatMasker annotations [39]. Variant reads were manually inspected using the Integrative Genomics Viewer (IGV) [40] to exclude any likely false positive variants with insufficient evidence or insufficient read coverage, consistent with recommended best practices for reducing false-positive variant calls in clinical sequencing [41, 42]. Variants with a heterozygous genotype call and a variant allele fraction of less than 33% or greater than 66%, variants with < 20X coverage, and variants with many mismatched bases in nearby reads were excluded. ClinVar [43, 44] was used to search for any pre-existing classifications or other variants occurring at the same nucleotide or amino acid position.

#### CNV classification for pathogenicity

For deletions and duplications identified by CNV and/or SV callers, automatic filters were applied and variants were retained if they met the following criteria: (i) were absent from or present at 1% frequency or less in a database of CNVs/SVs, generated from Illumina HiSeq X sequencing data of parents of children with autism spectrum disorder at TCAG [45] and called using the same methodology, (ii) variants that overlapped with an exonic region, and (iii) overlapped with a gene in the CHD gene list (with the exception of de novo variants which were assessed even if they did not overlap a CHD gene). To reduce the number of false-positive CNVs, only variants called by both ERDS and CNVnator were retained. Each variant that passed these automatic filters was queried through the DECIPHER browser [46]. Variants that overlapped with benign/population variants as seen in at least 10 individuals in DGV [47] or gnomAD structural variants [48] were not further considered, depending on the suspected mode of inheritance. The remaining variants were visualized using either IGV [40] or Samplot [49], depending on their size and complexity, to confirm their authenticity.

To visually determine if a variant was a true positive, the read depth, insert size, and orientation of paired reads in IGV or Samplot were assessed. Variants of interest were required to be associated with a noticeable drop (for deletions) and increase (for duplications) in coverage compared to the surrounding region to pass visual inspection. Coloring alignments by insert size in IGV was used to recognize differences in expected versus observed insert size which was helpful in detecting deletions and insertions. Orientation of paired reads was used as evidence to support structural variants such as inversions. True inversions were accounted for by the presence of cyan (forward) and purple (reverse) reads on both breakpoints.

Variants that passed the above inspections proceeded to manual ACMG/AMP classification [30]. Intragenic CNVs/SVs were submitted to AutoPVS1 [50], to automatically assign a PVS1 criterion for haploinsufficient genes (i.e., genes with a probability of being loss-of-function intolerant (pLI) score greater than or equal to 0.9, a ClinGen dosage curation indicating haploinsufficiency, and/or literature evidence supporting a loss-of-function pathogenic mechanism). Complex variants (called as dual DUP-DEL, DUP-DEL-INV, etc.) were only considered if there was phenotype support for the genes harboring them. Literature and databases such as OMIM and ClinVar were surveyed to identify similar CNVs/SVs reported in individuals with the phenotype of interest. Parental genome read alignments, if available, were visualized together with the proband, when determining variant inheritance (i.e., whether the variant was de novo, parentally inherited, or unknown), for the aforementioned ACMG/AMP guidelines.

All putative pathogenic/likely pathogenic protein-coding variants that were identified in GS data, but which had not been previously reported upon clinical genetic testing, were evaluated by our return of results committee [51] and were subsequently re-confirmed using clinical genetic testing.

### Identification of putatively splice-disrupting variants in GS data

Putatively splice-disrupting SNVs and indels were identified using SpliceAI [52], the tool recommended by the ClinGen Sequence Variant Interpretation Subgroup [13]. Exome annotations and splice junctions were similarly obtained from SpliceAI [52]. Pre-computed masked SpliceAI delta scores were utilized where possible (https://basespace.illumina.com/projects/66029966); otherwise, SpliceAI (v1.3.1) was used to generate masked delta scores with a maximum distance of 100 bp between the variant and gained/lost splice site. SNVs and indels with a “PASS” flag were extracted using bcftools v1.9 [53]. Variants with a SpliceAI delta score ≥ 0.2 were retained and subsequently annotated with the predicted effect (VEP v102 [54]), reported pathogenicity (ClinVar 2022–04-03 and HGMD Pro 2019), control allele frequency (gnomAD v2.1.1 and v3.1.2), gene constraint (gnomAD v2.1.1), genomic low complexity regions (https://github.com/lh3), genomic RepeatMasker regions (https://www.repeatmasker.org), and wild-type splicing branchpoints [55, 56]. Select variants were manually annotated with CADD-Splice PHRED scores [57], in order to assess the correlation between those scores and those derived by SpliceAI. Ensembl RNA transcripts were further annotated as canonical by MANE v1.0 (MANE Select or MANE Plus Clinical) [37]. These variants were then analyzed in corresponding myocardial RNA-Seq data from the same patient to determine if they were associated with splicing events.

### Identification of aberrant splicing events in myocardial RNA-Seq

#### Myocardial RNA-Seq

To detect aberrant splicing events at the tissue level, RNA-Seq was performed on ventricular myocardial samples available from 154 unrelated TOF probands and 43 unrelated cardiomyopathy probands from SickKids Heart Centre Biobank. In patients who had consented to biobanking, myocardium was obtained from leftover tissue at the time of cardiac surgery and was immediately snap-frozen in the operating room and stored in liquid nitrogen. Among CHD cases, the median age at surgery was 0.5 (range 0.1–14.3) years. None of the patients received inhibitors of nonsense-mediated decay prior to tissue resection. Total RNA was extracted from myocardial samples using the RNeasy Mini kit (QIAGEN, Canada). RNA samples were sent to TCAG (The Hospital for Sick Children, Toronto) for ribosomal RNA depletion and library preparation using the Illumina Stranded Total RNA Prep Ligation with Ribo-Zero Plus, and sequenced using Illumina HiSeq 2500 or NovaSeq platforms to generate paired end reads of 150 bases. Raw sequencing reads were trimmed by Trimmomatic v0.36 [18] for quality trimming and adapter clipping. The remaining reads were aligned to the GRCh37 reference genome (1000 Genomes Project reference genome, hs37d5) using STAR (v2.6.1.c) [58] with basic two-pass mode and Ensembl GTF (release version 87) [59] was used for the annotation. Gene and transcript expression level quantification were prepared using RSEM (v1.2.22) [60].

#### Identification of aberrant splicing events in myocardial RNA-Seq data

Aberrant splicing events were identified in RNA-Seq data using FRASER v1.8.1 [61]. Introns with unreliable detection were filtered out using the “filterExpressionAndVariability” method with default parameters except for requiring a minimal read count of 15 in at least one sample. An “AE” beta-binomial denoising autoencoder was used to fit the splicing models, with hyperparameters ψ_5_ = 14, ψ_3_ = 11, and θ = 5 for the *Discovery* cohort, ψ_5_ = 6, ψ_3_ = 5, and θ = 2 for the *CHD Validation* cohort, and ψ_5_ = 5, ψ_3_ = 4, and θ = 2 for the *cardiomyopathy Validation* cohort. Cohorts utilized distinct hyperparameters due to their different sizes and properties. Optimal autoencoder hyperparameters for each cohort were determined using the “optimHyperParams” method. Splice events were annotated using biomaRt as part of the “annotateRanges” method [62]. Observed events were considered to be significant with a false discovery rate < 0.2, an absolute *Z*-score ≥ 1, an absolute Δψ/θ score ≥ 0.2, and ψ/θ − Δψ/θ ≤ 0.1 or ≥ 0.9. Events annotated as not mapping to a gene or to multiple genes were excluded. All reported splicing events in CHD genes were visually inspected. A large number of RNA splicing outliers annotated as being in *MYH6* or between *MYH6* and *MYH7* were excluded, despite *MYH6* being a Tier 1 autosomal dominant CHD gene. *MYH6* and *MYH7* are adjacent in the genome, and these two genes share highly homologous exons. Visual inspection of the anomalous RNA-Seq data demonstrated that reads were often being aligned between homologous *MYH6*-*MYH7* exon junctions, rather than being true splicing outliers, and were therefore excluded as likely false-positives.

#### Identification of gene expression outliers in myocardial RNA-Seq data

As aberrant RNA splicing may result in nonsense-mediated decay, gene expression outliers in RNA-Seq data were identified using OUTRIDER (v1.8.0) [63]. OUTRIDER was run on the *Discovery* (TOF), CHD *Validation*, and cardiomyopathy *Validation* cohorts separately. Low-expressed genes were first filtered out by only selecting genes with at least 10 read counts in more than 50% of the input samples in each cohort. Before fitting the input cohort to the OUTRIDER model, the optimal encoding dimension "q" was first determined by using the "findEncodingDim" method. The optimal encoding dimension was estimated to be 16 for the CHD *Discovery* cohort, 8 for the CHD *Validation* cohort, and 8 for the cardiomyopathy *Validation* cohort. To further identify whether variants were associated with reduced RNA expression, we used the OUTRIDER *Z*-scores calculated across each gene.

### Generation and validation of random forest models for selecting heart-specific splice-disrupting variants

To identify true-positive splice disrupting variants, genome-wide variants were classified by whether or not they were associated with confirmed splicing events in the myocardium by searching for matching significant events within ± 100 bp of altered splicing boundaries called by FRASER. Variants associated with significantly altered outlier splicing in the same gene, but occurring outside of these boundaries were deemed indeterminate and were excluded from model training and validation. To test for the enrichment of univariable features that associate with variants that validated by FRASER, we used two-sided Mann–Whitney *U* tests for continuous variables and two-sided Fisher’s exact tests for binary variables. The R package randomForest (v4.7–1.1) [64] was used with default parameters to create and test four machine learning models for the prediction of splice-disrupting variants. Model 1 used only SpliceAI Δ scores as input; model 2 included DNA variant features associated with tissue splicing events, i.e., the variant distance to the nearest annotated splice junction, the variant type (SNV or indel), and whether the variant occurred in a branchpoint region, low complexity region, and/or repetitive region, while excluding SpliceAI Δ scores; model 3 included all of the DNA variant features from model 2 in addition to the corresponding median gene expression TPM value in RNA-Seq data, and model 4 included the maximum SpliceAI Δ score as well as additional DNA variant features and gene expression TPM values. Variants with missing feature values were omitted by the models (i.e., missing values were not imputed). To account for the imbalance in the training class frequencies, training models were either inversely weighted by the corresponding number of observations in the training data (Weighted models), or used Synthetic Minority Over-sampling Technique (SMOTE models) to artificially create new training inputs of the minority positive (variant resulting in confirmed splice-disruption) class [65]. All models were internally evaluated for performance by area under the curve (AUC), sensitivity, specificity, odds ratios, and Fisher’s exact test *p*-values using internal five-fold cross-validation. To reduce bias in odds ratios calculations and to avoid “zero cells” in the contingency tables, 0.5 was added to each observed cell frequency (Haldane-Anscombe correction). Bias-variance–covariance decomposition analysis was performed on binary classifications using the R library “randomUniformForest” (v1.1.6). The models were subsequently retrained on the entire *Discovery* cohort prior to its application to additional cohorts.

#### Validation of model performance in independent CHD and cardiomyopathy cohorts

In order to independently assess the performance of random forest models for selecting splice-disrupting variants, all models were applied to two independent cohorts of CHD (*n* = 48) and cardiomyopathy (*n* = 43) cases. Putative splice-disrupting variants in these samples were identified and annotated as described above. Variants were further limited to those found in only one sample in each cohort (i.e., an internal minor allele frequency (MAF) < 0.03), as only internally rare variants were expected to be associated with outlier splicing events. Each variant was annotated by whether or not it was associated with a confirmed splicing event in the myocardium as detected by FRASER, and whether or not it was selected by each model. Model performance was evaluated within each validation cohort by AUC, sensitivity, specificity, odds ratios, and Fisher’s exact test *p*-values. In addition, in order to determine whether putative splice-disrupting variants led to nonsense-mediated decay, gene expression *Z*-score values were obtained using OUTRIDER for each associated sample harboring a variant in the gene. The values were stratified for variants selected by each model versus those rejected by the model, and then compared using two-sided *t*-tests. The weighted model 4, which validated as having optimal performance, was then applied to the remainder of the CHD cohort to identify additional high-confidence splice-disrupting variants.

### Identification of high-confidence splice-disrupting variants in extended CHD cases and controls using the optimal random forest model

The optimal random forest weighted model 4 was applied to all GS data from the *Extension* and *Control* cohorts in order to identify high-confidence splice-disrupting variants. Variants were further filtered to include only those rare in gnomAD control populations [38] (gnomAD v2 allele frequency < 0.0001, and gnomAD v3 PopMax allele frequency at 95% confidence < 0.0001). Both gnomAD v2 and v3 statistics were used in order to account for differences in the reference genomes used to align the *Extension* (hg38) and *Control* (GRCh37) cohorts). Variants in CHD genes in the *Extension* cohort were subsequently visually inspected. Six putative splice-disrupting indels in *SMAD4* found in two TOF probands were subsequently excluded. The DNA reads associated with these variants had poor alignments upon visual inspection, and further investigation identified a report of a rare pseudogene containing homologous *SMAD4* exons, which is found in < 1% of the population [66], suggesting that these DNA variants were false positives. Alignment files for control samples were not available, and thus variants in this cohort were not visually confirmed or otherwise excluded.

#### Gene set enrichment analysis

To identify which phenotypic abnormalities in human disease were enriched for splice-disrupting variants, enrichment analysis of gene sets harboring 133 high-confidence splice-disrupting variants in CHD genes among all CHD probands (*n* = 1101) was performed using g:Profiler core tool g:GOSt [67, 68]. Phenotypic abnormality gene sets were obtained from the Human Phenotype Ontology [69]. We limited the search to a custom background gene set containing all genes annotated by Matched Annotation from NCBI and EMBL-EBI (MANE) [37], that were expressed in the RNA-Seq profiles derived from patient myocardium in the Discovery cohort (median TPM ≥ 1), and that had at least one annotation in Human Phenotype Ontology gene sets. Genes were input as symbols derived from SpliceAI annotations. An adjusted *p*-value threshold of 0.01 was used to determine significance of all gene sets. Adjusted *p*-values were calculated using the g:SCS (Set Counts and Sizes) method which considers dependencies between multiple tests by taking into account the overlap in functional terms [67, 68].

#### Case–control burden analyses

To directly compare the burden of short variants in cases and controls, we first determined whether technical differences between cases and controls (e.g., sequencing facility, GS platform, reference genome version, variant detection workflows) contribute to a systematically different burden of synonymous variants genome-wide. Our strategy to filter for rare synonymous variants in samples was similar to how we had identified high-confidence splice-disrupting variants. We limited to only SNVs and indels with a “PASS” flag, and then annotated variants using VEP v102 [54]. Variants were filtered to only include those with an internal minor allele frequency (MAF) < 0.01, gnomAD v2 allele frequency < 0.0001, and gnomAD v3 PopMax allele frequency < 0.0001 [38]. Both gnomAD v2 and v3 statistics were used in order to account for differences in the reference genomes used to align the *Extension* (hg38) and *Control* (GRCh37) cohorts, and internal allele frequencies were used to further reduce possible false-positive variant calls. We calculated for each sample the number of rare variants predicted to result in a synonymous substitution. Protein-coding regions of the genome were filtered using the UCSC Table Browser [70] for assembly hg38 with the GENCODE v44 track [71], and limited to genes in autosomal chromosomes 1–22. Synonymous variants were further required to be annotated as such in a MANE canonical transcript [37]. The burden of synonymous variants between cases and controls was derived using a Mann–Whitney *U* test to compare the continuous allele frequency of variants across samples. For burden analysis of splice-disrupting variants between cases and controls, we identified the set of rare SNVs and indels across all samples that were selected by the optimal machine learning model as being high-confidence splice-disrupting variants. For case–control burden of splice-disrupting variants, *p*-values were calculated using a two-sided Fisher’s exact test, comparing the number of samples in each cohort that harbored at least one high-confidence splice-disrupting variant versus those that harbored none. To reduce bias in odds ratios calculations and to avoid “zero cells” in the contingency tables, 0.5 was added to each observed cell frequency (Haldane-Anscombe correction).

### Data analyses and visualizations

All aforementioned statistical analyses, as well as data visualizations, were carried out using the R Programming Environment v4.1.2. Graphical data plots were created using the ggplot2 [72] and pROC [73] libraries.

## Results

### Study cohort

The overall study included 1101 CHD probands, of which 875 had TOF and 226 had TGA (Table S1) [16]. Probands with a clinically and/or genetically diagnosed syndrome were excluded. However, those who had extra-cardiac features without a syndromic diagnosis, i.e., unexplained genetic etiology at the time of enrolment were retained (11% of the cohort). Cardinal syndromic features may not always be evident in infancy and therefore “syndromes” cannot be excluded based on clinical phenotype alone in young children. CHD probands were subdivided into a *Discovery* cohort (*n* = 106), a CHD validation cohort of 48 unrelated TOF probands (*n* = 48) and a second validation cohort of cardiomyopathy probands (*n* = 43), all of whom had both GS and myocardial RNA-seq. CHD probands (721 TOF and 226 TGA) with only GS data constituted an *Extension* cohort (*n* = 947). Eighteen percent of probands in the *Discovery*, *Validation*, and *Extension* cohorts received one or more forms of prior clinical genetic testing, including cytogenetic, microarray, single-gene polymerase chain reaction, gene panel, and/or exome sequencing, of which 2% harbored pathogenic or likely pathogenic protein-coding variants (accounting for < 1% of the entire cohort − < 1% TOF and 0% TGA). Two thousand five hundred seventy GS samples from the Medical Genome Reference Bank [17] were obtained for use as a Control cohort.

### Protein-coding variants in CHD genes

We first analyzed GS data to identify additional pathogenic or likely pathogenic variants in CHD-associated genes (Table S2). ACMG/AMP criteria [29, 30] were applied to protein-coding (non-splicing) SNVs, indels, and CNVs in CHD genes, yielding pathogenic variants in 4% of these probands (4% TOF and 2% TGA) (Table S1).

### Discovery of splice-disrupting variants affecting cardiac-expressed genes

We explored GS data in the *Discovery* cohort in which both GS and myocardial RNA-Seq data were available (*n* = 106) for DNA SNVs and indels that were predicted with high sensitivity using SpliceAI [52] to result in the loss of a wild-type splice junction and/or the gain of a novel cryptic splice site. A SpliceAI Δ score ≥ 0.2 was used as a screening cut-off to maximize variant detection. We exclusively utilized SpliceAI to detect putative splice variants, as this was the tool recommended by the ClinGen Sequence Variant Interpretation Subgroup [13]. This identified 17,528 variants of which 8583 were rare within the *Discovery* cohort, i.e., found in no more than one sample (internal MAF < 0.01). Among these, 386 (4%) occurred at canonical splice sites. To determine which splice-disrupting variants were associated with corresponding aberrant splicing events in the myocardium, we first searched for all splice-disrupting events in patient myocardium by applying the in silico tool FRASER [61] to myocardial RNA-Seq data (*n* = 106) which allows detection of not only alternative splicing but also intron retention events [61]. Across all RNA-Seq samples, 11,540 MANE-annotated genes had a median TPM expression ≥ 1, i.e., were expressed in cardiac tissue. We limited our selection of splice sites to those where RNA reads from the donor and acceptor sites were aligned within the same gene, and either rarely were observed to be spliced together within the cohort (ψ/θ − Δψ/θ ≤ 0.1) or nearly always were observed to be spliced together (ψ/θ − Δψ/θ ≥ 0.9). Significant outlier splicing events within a sample were then defined as those having a false discovery rate < 0.2, an absolute *Z*-score ≥ 1, and an absolute Δψ/θ score ≥ 0.2, indicating that alternative splicing between two sites was observed 20% more or less often than expected. This yielded 844 significantly altered genes with affected splice junctions and/or intron-retention events with a high effect size. A median of 6 genes were affected per sample, with 134 genes altered in more than one patient. The number of significant outlier splicing events in each sample was not associated with patient age at surgery (*p* > 0.05).

**Fig. 1:**
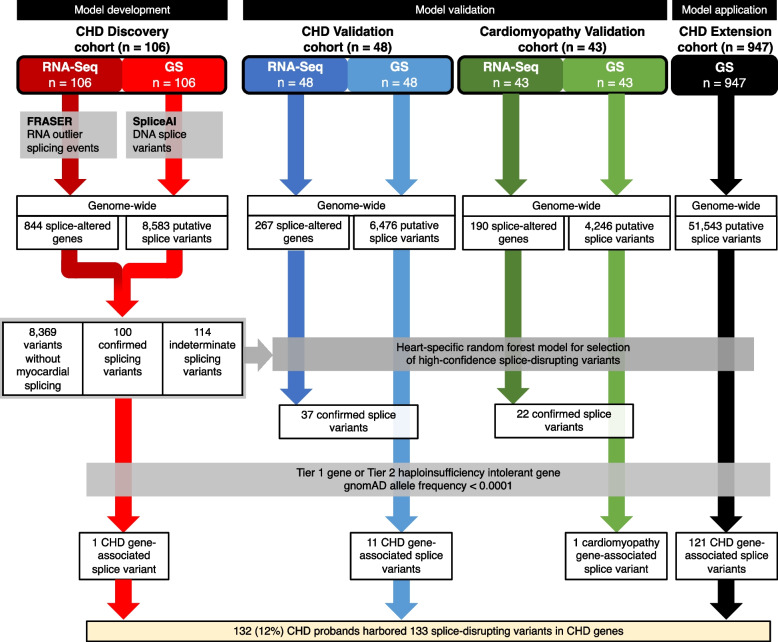
Schematic workflow for development, validation, and application of a random forest model for selecting high-confidence splice-disrupting variants for congenital heart disease. Selection strategy is shown for the identification of splice-disrupting variants in CHD genes. *Model development*: CHD Discovery cohort (*n* = 106) was used to identify putative splice-disrupting variants in genome sequencing (GS) data and confirm whether the variants were associated with a significant effect in RNA-sequencing (RNA-Seq) data derived from patient myocardium. These variants and their confirmed effect were then used to construct random forest models for predicting splice-disrupting variants with high-confidence. *Model validation*: Model performance was validated using independent CHD validation (*n* = 48) and cardiomyopathy validation (*n* = 43) cohorts, where both GS and RNA-Seq profiles were available for all probands. *Model application*: The optimal random forest model was applied to a CHD Extension cohort (*n* = 947), where only GS data were available for all probands. One hundred thirty two (12%) CHD probands harbored 133 rare, high-confidence splice-disrupting variants in CHD genes, including 47 variants in Tier 1 CHD genes and 86 variants in Tier 2 haploinsufficiency-intolerant CHD genes. RNA-Seq, RNA sequencing; GS, genome sequencing; FDR, false discovery rate; MAF, minor allele frequency; CHD, congenital heart disease

**Fig. 2:**
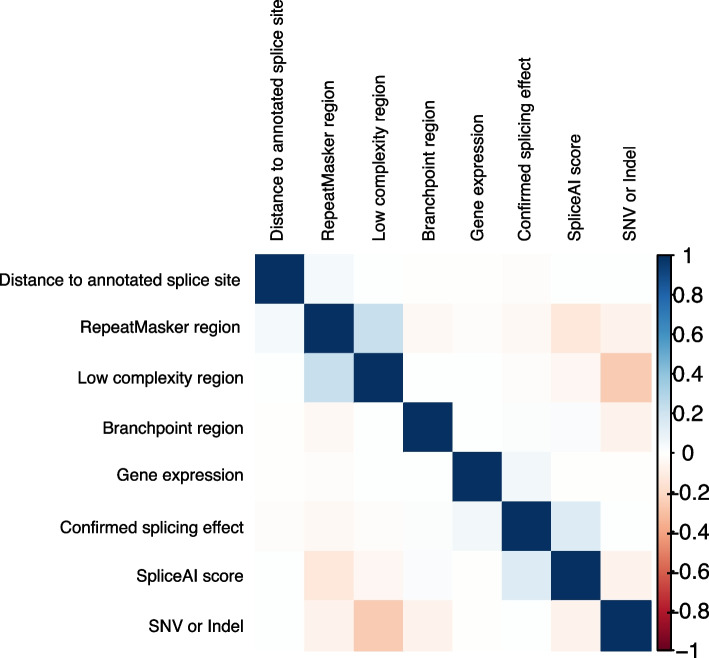
Correlation matrix for DNA variant features used in model development. The matrix shows minimal correlation between DNA variant input features used in developing random forest models in the *Discovery* cohort

We then classified the rare DNA variants identified in GS data by whether or not they were associated with a matching significant outlier splicing event in myocardial RNA-Seq data from the same proband. This yielded 100 DNA variants in 95 genes that were associated with observable splice disruption in the myocardium, i.e., confirmed splicing events (Table S3), and 8369 DNA variants where a significant tissue effect was not observed. One hundred fourteen DNA variants were excluded due to an indeterminate association with an observed splicing event, i.e., more than 100 bp outside of a significantly altered splice donor/acceptor pair. Only 33 (33%) of the confirmed splicing events occurred at canonical splice sites. An overview of our variant prioritization strategy is shown in Fig. 1. A comparison of confirmed splice-disrupting variants, i.e., DNA variants associated with a splicing event in the myocardium, versus the remaining unconfirmed splice-disrupting variants revealed that true positive variants had higher SpliceAI Δ scores (p = 1.1 × 10^−22^), affected genes with higher RNA expression (*p* = 1.6 × 10^−24^), and were less likely to occur in repetitive (RepeatMasker) regions of the genome (*p* = 3.8 × 10^−4^). Variant features generally had low correlation with one another, though RepeatMasker and low complexity regions were positively correlated, whereas variants with high SpliceAI scores and SNVs were negatively correlated with RepeatMasker and low complexity regions (Fig. 2). Overall, this suggested that annotated variant DNA features independently contributed to the prediction of true splice-disruption. Although we did not compare multiple published tools for the identification of putative splice variants, among confirmed variants within our Discovery cohort we observed that the maximum SpliceAI Δ score and the CADD-Splice PHRED score were significantly correlated (*p* = 1.11 × 10^−9^), indicating that these two methodologies provide comparable statistics. Of note, all confirmed variants were in genes with a median TPM expression > 0.5 in our cohort. Among all putative splice-disrupting variants a (SpliceAI Δ score), a median TPM expression score threshold of 0.5 yielded a sensitivity of 0.65 and specificity of 0.78, while a threshold of 0.8 yielded a sensitivity of 0.47 and specificity of 0.92.

### Random forest model to predict cardiac relevant non-canonical splice-disrupting variants

**Fig. 3:**
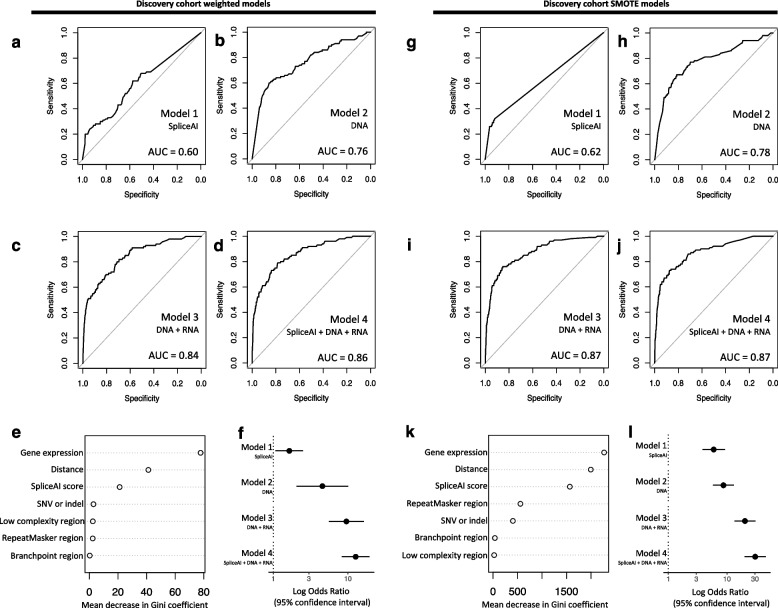
Performance of random forest models for splice-disrupting variants on internal cross-validation. Four types of models each were designed using either class weights or SMOTE to address class imbalance; internal performance was assessed using five-fold cross-validation to compare area under the curves (AUC) for each model. *Weighted model performance*: **a** SpliceAI only AUC, **b** DNA variant features only AUC, **c** DNA variant features + myocardial RNA gene expression AUC, **d** SpliceAI + DNA variant features + myocardial RNA gene expression AUC. **e** Gini coefficient showing the importance of a specific feature to the nodes and leaves of the random forest model 4. **f** The odds ratio for selecting variants confirmed to affect splicing was highest for model 4. *SMOTE model performance*: **g** SMOTE SpliceAI only AUC, **h** DNA variant features only AUC, **i** DNA variant features + myocardial RNA gene expression AUC, **j** SpliceAI + DNA variant features + myocardial RNA gene expression AUC. **k** Gini coefficient showing the importance of a specific feature to the nodes and leaves of the random forest model 4. **l** The odds ratio for selecting variants confirmed to affect splicing was highest for model 4

Utilizing features from the set of confirmed splicing events, we trained random forest models to predict whether variants identified in GS data are associated with aberrant splicing in human myocardium. Due to the imbalance between the number of confirmed versus unconfirmed putatively splice-disrupting variants, trained models were either weighted to prioritize the selection of the confirmed class, or utilized the Synthetic Minority Oversampling Technique (SMOTE) to artificially create a balanced training set [65]. Model 1 included only SpliceAI Δ scores as input. While SpliceAI has been reported to have good accuracy for detecting splice-disrupting variants, it utilizes only the genomic sequence of pre-mRNA transcript as input, which does not take into account existing splice junction boundaries or the likelihood of false positive variant calls. We therefore trained a second model 2 which included the DNA variant features associated with true splicing, i.e., variant distance to the nearest annotated splice junction, the variant type (SNV or indel), and whether the variant occurred in a branchpoint region, low complexity region, and/or repetitive region, while excluding SpliceAI Δ scores. As these two models included only DNA variant features, they are not trained to predict organ-specific splicing validation that may be unique to cardiac-expressed genes. A third model 3 was thus trained and included the aforementioned DNA variant features from model 2 in addition to the corresponding median gene expression TPM value in patient myocardial samples. Finally, a fourth model 4 included all the above, i.e., the DNA variant features, myocardial gene expression values, and SpliceAI Δ scores. The performance of all four models using each training approach was internally assessed using five-fold cross validation. While all four models performed better than random, the models that included DNA variant features and/or DNA variant features with cardiac gene expression values showed better model performance than the SpliceAI model alone. Overall, model 4, which included all DNA variant features and gene expression values along with SpliceAI scores, provided highest performance accuracy on five-fold cross validation. This was observed using weighted- and using SMOTE-trained approaches (five-fold cross-validation AUC = 0.86 and 0.87, respectively) (Fig. 3d and j). Although the weighted model 4 prioritized gene expression values, SpliceAI Δ scores and distance from the nearest existing annotated splice site also provided independent predictive information (Fig. 3e and l). Importantly, regardless of the base methodology used to address class imbalance, adding additional DNA features and heart-specific information improved model performance for selecting cardiac-relevant high-confidence splice disrupting variants. A bias-variance decomposition analysis similarly demonstrated that model 4 had the smallest mean squared error and squared bias (Table S4). Although the weighted class and SMOTE-trained models both had high AUC values, the weighted models had superior sensitivity to select for confirmed splicing variants (0.69 and 0.62 on internal five-fold cross validation, respectively for model 4). We therefore used the weighted-trained model 4 to identify high-confidence splice-disrupting variants in the CHD *Validation* and *Extension* cohorts.

### Independent validation of the random forest model in CHD and cardiomyopathy cohorts

**Fig. 4:**
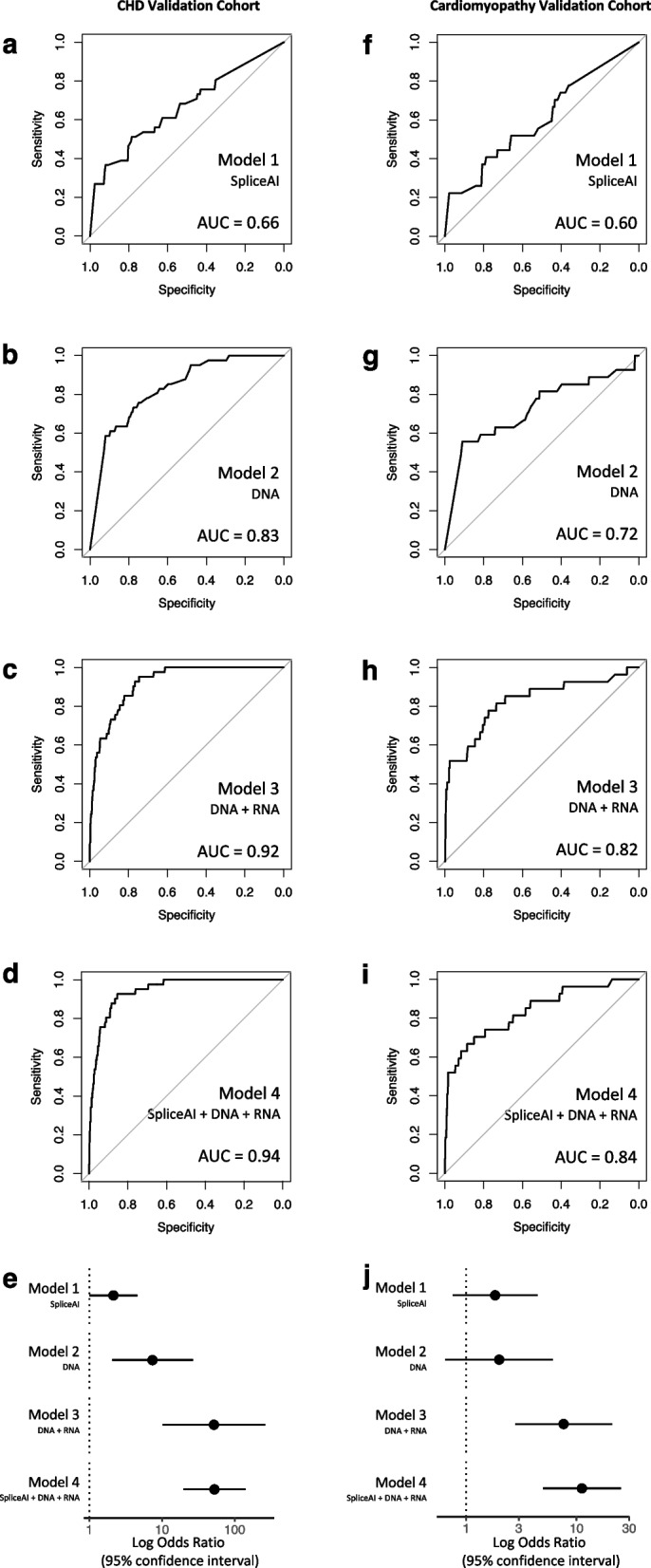
Performance of weighted random forest model for splice-disrupting variants applied to external validation cohorts. The performance of the weighted model was assessed in two external validation cohorts. *CHD validation cohort*: **a** SpliceAI only AUC, **b** DNA variant features only AUC, **c** DNA variant features + myocardial RNA gene expression AUC, **d** SpliceAI + DNA variant features + myocardial RNA gene expression AUC. AUC was highest for model 4 in CHD cohort (*n* = 48). **e** The odds ratio for selecting variants confirmed to affect splicing was highest for model 4 in CHD cohort. *Cardiomyopathy validation cohort*: **a** SpliceAI only AUC, **b** DNA variant features only AUC, **c** DNA variant features + myocardial RNA gene expression AUC, **d** SpliceAI + DNA variant features + myocardial RNA gene expression AUC. AUC was highest for model 4 in cardiomyopathy cohort (*n* = 43). **j** The odds ratio for selecting variants confirmed to affect splicing was highest model 4 in cardiomyopathy cohort

In order to externally validate the performance of the random forest model, we applied the model to two independent *Validation* cohorts of 48 CHD probands and 43 cardiomyopathy probands, for whom both GS and matching RNA-Seq profiles derived from the right or left ventricular myocardium, respectively, were available [6]. We first selected SNVs and indels that were predicted with high sensitivity to result in the loss of a wild-type splice junction and/or the gain of a novel cryptic splice site (SpliceAI Δ score ≥ 0.2), and that were rare within the *Validation* cohorts, i.e., found in no more than one sample (MAF < 0.03). Within the CHD and cardiomyopathy *Validation* cohorts this identified 6476 and 4246 DNA variants genome-wide, of which 908 and 727 were selected by the weighted random forest model 4, respectively. We next identified aberrant splicing events in the *Validation* cohorts using FRASER. Using the previous thresholds (false discovery rate < 0.2, an absolute *Z*-score ≥ 1, an absolute Δψ/θ score ≥ 0.2, and ψ/θ − Δψ/θ ≤ 0.1 or ≥ 0.9) yielded 430 and 360 observations, respectively, of significantly-altered splicing events with a high effect size. We applied all random forest models to these data and then to further ensure independence we excluded variants that were identically shared between the *Discovery* and *Validation* cohorts. This showed that, similar to the *Discovery* cohort, model 4 outperformed all other models, with an AUC of 0.94 and 0.84 in the CHD and cardiomyopathy *Validation* cohorts, respectively (Fig. 4). DNA variants with confirmed altered splicing were significantly enriched among those selected by model 4 compared with variants without a confirmed splicing effect (*p* = 1.3 × 10^−27^ and *p* = 1.6 × 10^−9^, CHD and cardiomyopathy *Validation* cohorts, respectively). Confirmed splicing variants, as well as all variants selected by model 4, are included in Table S5, while contingency tables and performance metrics, including a bias-variance decomposition analysis, are included in Table S6. We additionally investigated whether selected splice-disrupting DNA variants were associated with a reduction in gene expression, which may occur due to nonsense-mediated decay. Applying OUTRIDER [63] to the *Validation* cohorts, we indeed observed that samples harboring DNA variants selected by model 4 had lower corresponding gene expression *Z*-scores compared with samples harboring variants that were not selected by the random forest model (mean *Z*-score of − 0.22 versus − 0.049 respectively in the CHD *Validation* cohort, *p* = 0.00040; mean *Z*-score − 0.23 versus − 0.049 respectively in the cardiomyopathy *Validation* cohort, *p* = 0.00072). Of note, the weighted model 4 selected a pathogenic canonical splice site variant in a known cardiomyopathy gene, *FLNC*, previously reported by our group to be associated with reduced mRNA expression despite only a few RNA-Seq reads displaying abnormal splicing (presumably as a result of nonsense-mediated decay) [6]. This variant was confirmed in a clinical testing laboratory and deemed to be disease-causing by our return of results committee and the clinical testing lab [51]. Together these results confirm that our cardiac-specific random forest model improved the selection of true splice-disrupting variants in patients with childhood onset heart disease.

### High-confidence splice-disrupting variants in CHD genes in the CHD Extension cohort

#### Splice-disrupting variants in Tier 1 CHD genes

**Fig. 5:**
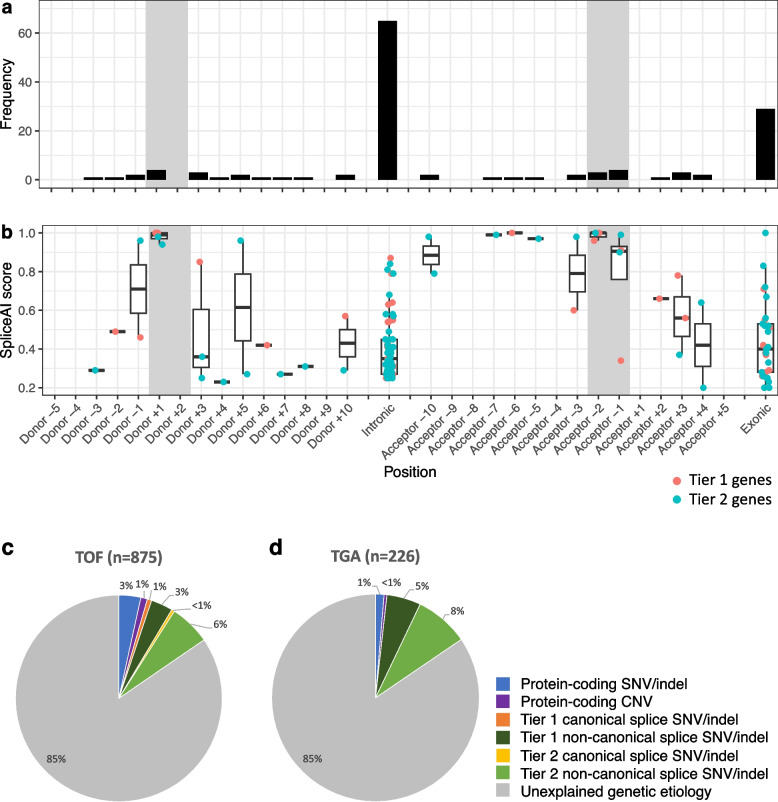
Frequency of high-confidence splice-disrupting variants in CHD genes. One hundred thirty three confirmed and high-confidence splice disrupting variants in CHD genes were identified in the 1101 CHD patients—Discovery (*n* = 106), Validation (*n* = 48), and Extension (*n* = 947) cohorts. Variants were mapped to their closest annotated wild-type splice site within their corresponding gene. Canonical splice regions are highlighted in gray. **a** *Variant position*: Intronic variants > 10 bp from a splice junction accounted for 49% of all variants. **b** *SpliceAI Δ variant scores*: Splice-disrupting variants showed high variability in SpliceAI scores. Putatively disease-causing splice-disrupting variants in Tier 1 CHD genes were found in **c** 4% of TOF probands, and **d** 5% of TGA probands without an explained genetic etiology, with non-canonical variants representing a majority of splice disrupting variants. TOF, tetralogy of Fallot; TGA, Transposition of the great arteries; SNV, single-nucleotide variant; indel, insertion-deletion

**Fig. 6:**
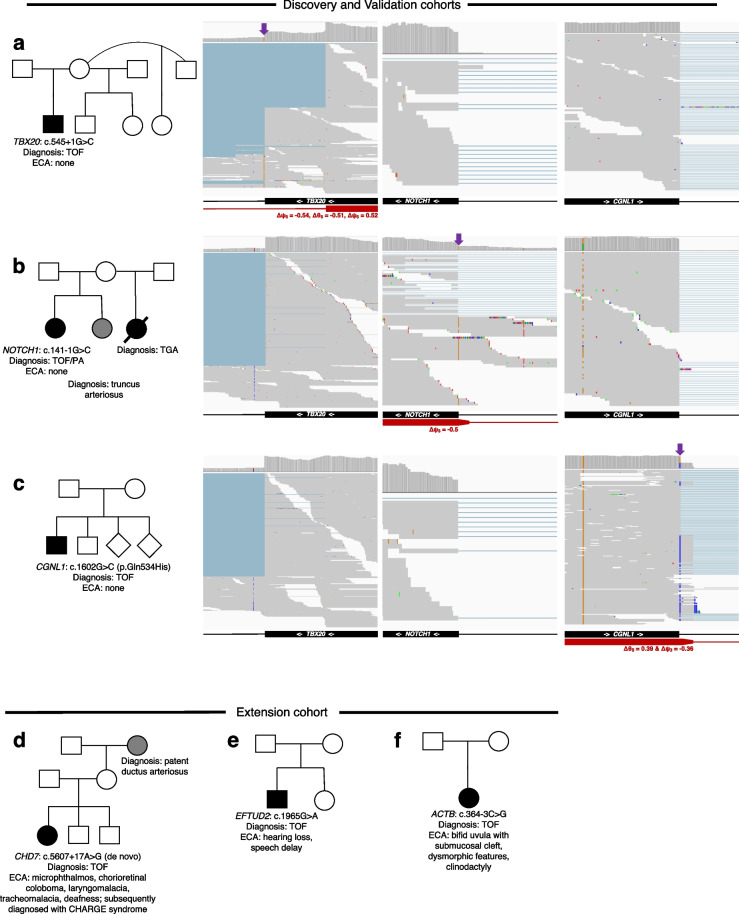
Representative splice-disrupting variants in CHD genes. Family pedigrees with CHD harboring representative high-confidence splice disrupting variants in Tier 1 and 2 genes are shown. **a** *TBX20* (Tier 1), **b** *NOTCH1* (Tier 1), **c** *CGNL1* (Tier 2), **d** *CHD7* (Tier 1), **e** *EFTUD2* (Tier 1), and **f** *ACTB* (Tier 2). Wild-type exon/intron boundaries below IGV screenshots of RNA-Seq data are represented in black, and alternatively observed boundaries are represented in red. FRASER statistics for outlier splicing events are written below the alternative boundaries. Arrows next to gene names represent reading direction. Purple arrows represent location of DNA splice-disrupting variant. TOF, tetralogy of Fallot; ECA, extracardiac anomalies

The random forest weighted model 4 was applied to a CHD *Extension* cohort of 947 patients. A total of 51,543 putative splice-disrupting variants were identified genome-wide (SpliceAI Δ score ≥ 0.2), of which the weighted model 4 selected 5658 variants. Filtering for variants that were rare in controls (gnomAD v2 allele frequency < 0.0001 and gnomAD v3 PopMax allele frequency < 0.0001) and annotated as being in intragenic regions of MANE transcripts yielded 4222 variants in 2819 genes, of which 2818 genes were highly expressed in myocardial-derived RNA-Seq samples in the *Discovery* cohort (median TPM ≥ 1). We further narrowed our variant selection by limiting variants to those in a Tier 1 CHD gene with either a dominant mode of inheritance or homozygous in a gene with a recessive mode of inheritance (Table S7). In total, across the CHD *Discovery*, *Validation*, and *Extension* cohorts, we identified 47 rare high-confidence splice-disrupting DNA variants in 49 probands involving 26 Tier 1 genes (Fig. 5). Only six variants were located at a canonical splice site, all of which were considered to be pathogenic/likely pathogenic by ACMG/AMP criteria. These included a splice donor variant in *TBX20* as well as a splice donor variant in *NOTCH1*, which were both validated by RNA-Seq (Fig. 6). Intronic variants more than 10 bp from existing splice sites accounted for 47% of high-confidence Tier 1 splice-disrupting variants, most of which would not be detectable through panel or exome sequencing and would have been missed by applying only a high SpliceAI score cut-off. Two deeply intronic DNA variants in as many genes were observed in multiple unrelated probands in the *Extension* cohort. Of note, only one of these probands (carrying a non-canonical splice variant in *GATA4*) harbored an additional pathogenic protein-coding variant in Tier 1 CHD genes (a missense variant in *PTPN11*) to explain their CHD (Table S7).

#### Splice-disrupting variants in Tier 2 CHD genes

In addition to splice-disrupting variants in Tier 1 CHD genes, we searched for rare, high-confidence splice-disrupting variants in haploinsufficiency intolerant Tier 2 CHD genes (gnomAD v2 pLI ≥ 0.9) in the CHD *Discovery*, *Validation*, and *Extension* cohorts. This search yielded 86 variants in 49 genes among 89 probands (Table S3, Table S5, and Table S7). Five of these variants were observed in multiple cases, and five of these probands harbored additional pathogenic protein-coding variants. The relative positions of these 86 variants were also consistent with what was observed in Tier 1 CHD genes, with deeply intronic variants (> 10 bp from an existing splice site) accounting for 50% of all Tier 2 variants. For 73 of 86 variants (85%), the most probable predicted effect was the creation of a new cryptic splice site rather than the loss of an existing splice junction.

**Fig. 7:**
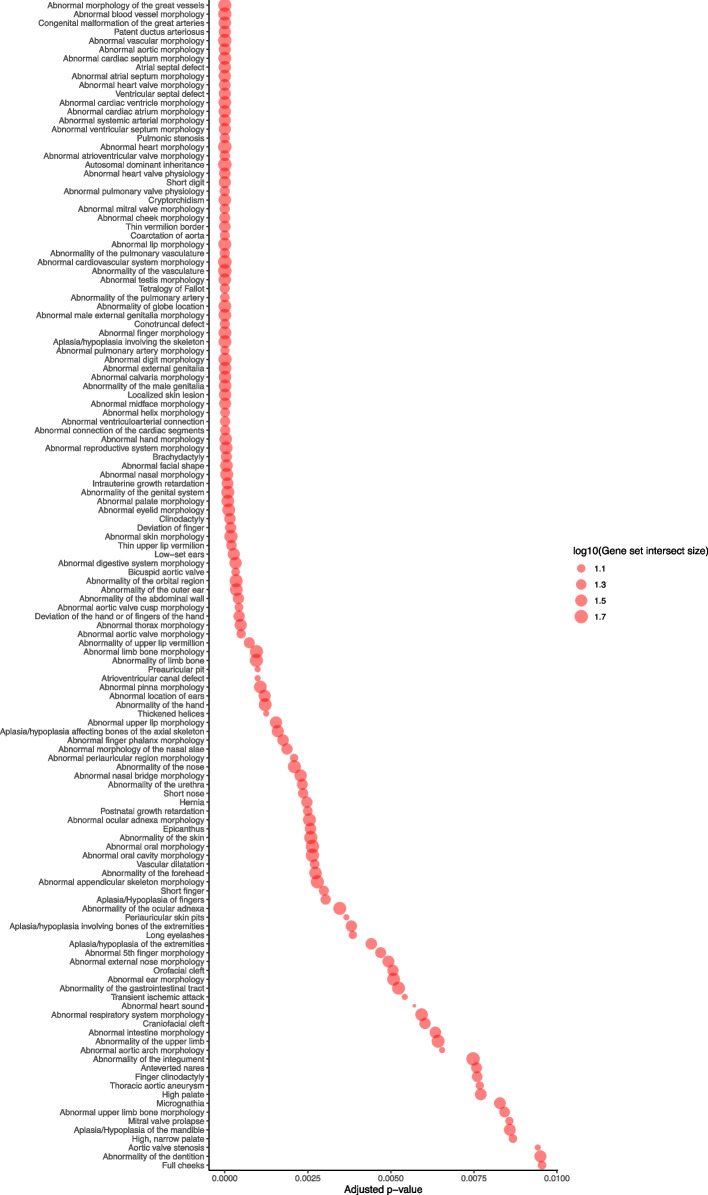
Gene sets enriched for splice-disrupting variants in CHD genes in the CHD *Discovery*, *Validation*, and* Extension* cohorts (*n* = 1101). Graph showing significantly enriched Human Phenotype Ontology (HP) terms among genes affected by high-confidence splice-disrupting variants in CHD genes

Across both Tier 1 and Tier 2 CHD genes in all CHD probands (*n* = 1101), 11/133 (8%) of high-confidence splice-disrupting variants occurred at canonical splice sites, and 65/133 (49%) were in deeply intronic regions (> 10 bp from an existing splice site). We observed that 15/133 (11%) of high-confidence variants were predicted to only disrupt existing splice sites, 107/133 (80%) were predicted to create a cryptic splice site without disrupting a canonical splice site, and the remaining 11/133 (8%) were predicted to have both effects (Table S3, Table S5, and Table S7). None of these variants were observed in branchpoint regions, although confirmed variants in branchpoint regions of non-CHD genes were observed in the *Discovery* and *Validation* cohorts. Among all genes expressed in the myocardial tissue of *Discovery* cohort cases (median TPM ≥ 1), the genes harboring these 133 rare, high-confidence splice-disrupting variants were enriched for a diverse set of Human Phenotype Ontology terms that included abnormal heart and vessel morphologies (Fig. 7, Table S8).

#### Genotype–phenotype correlation

We performed reverse phenotyping in probands harboring splice-disrupting variants in syndromic genes and identified some patients that harbored extra-cardiac features consistent with a syndrome even though it had not been clinically diagnosed at the time of the study. For example, within the *Extension* cohort, one patient had an intronic *CHD7* c.5607 + 17A > G variant that was de novo with the patient demonstrating features consistent with CHARGE syndrome (Fig. 6). Previous clinical testing for gene defects had been negative. Our study finding triggered repeat clinical genetic evaluation that led to confirmation of the genetic diagnosis of CHARGE syndrome. In another example, a proband with classic TOF harboring a cryptic splice acceptor gain variant in the protein-coding region of *EFTUD2* displayed partial clinical features of *EFTUD2*-associated syndrome including delayed speech, and mild to moderate hearing loss, likely of middle ear origin [74]. Another TOF proband harboring a splice region variant in the Tier 2 gene *ACTB* (c.364-3C > G) had extracardiac anomalies including bifid uvula with submucosal cleft, dysmorphic features, and clinodactyly, consistent with expected phenotype associated with this gene defect.

**Fig. 8:**
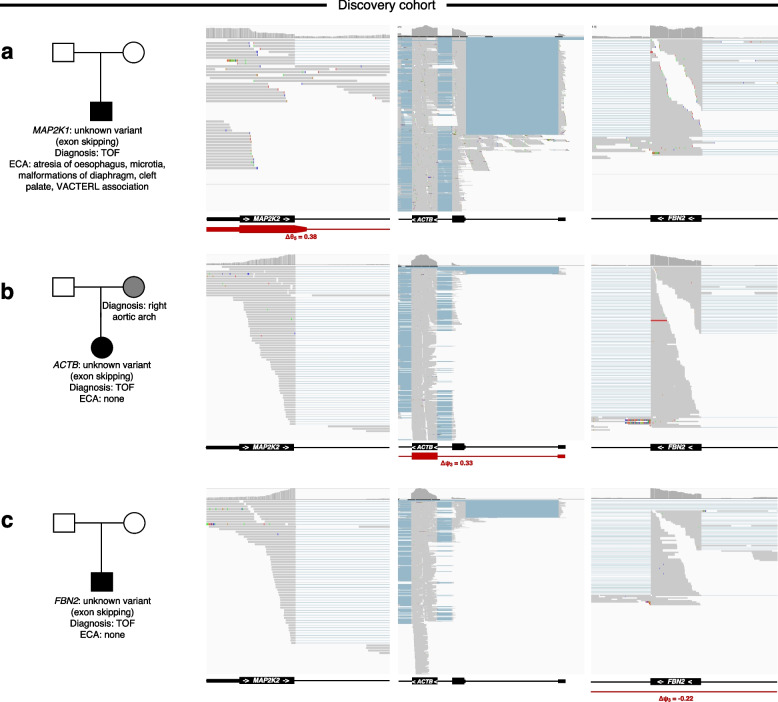
Altered splicing events in CHD genes in myocardium without an identified DNA variant. Family pedigrees with CHD showing representative aberrant splicing events in CHD genes without corresponding DNA variants are shown. **a** *MAP2K1* (Tier 1), **b** *ACTB* (Tier 2), and **c** *FBN2* (Tier 2). IGV screenshots of RNA-Seq data for all samples are shown TOF, tetralogy of Fallot; ECA, extra cardiac anomalies

One proband harbored significantly increased intron retention in RNA-Seq in the first exon of the *MAP2K1* gene, despite no candidate DNA variant being identified, including after searching for more common variants in the gene (Fig. 8). This individual was found to have congenital facial malformations on reverse phenotyping, which appeared to be consistent with cardiofaciocutaneous syndrome associated with this gene defect, and supports the pathogenicity of this alternative splicing event. It is noteworthy that a total of 27 significant splicing events in CHD genes were found without corresponding DNA variants (Fig. 8, Table S3, and Table S5). These events were not included in model development but remain candidates for further investigation.

### Burden of splice-disrupting variants in cases versus controls

**Fig. 9:**
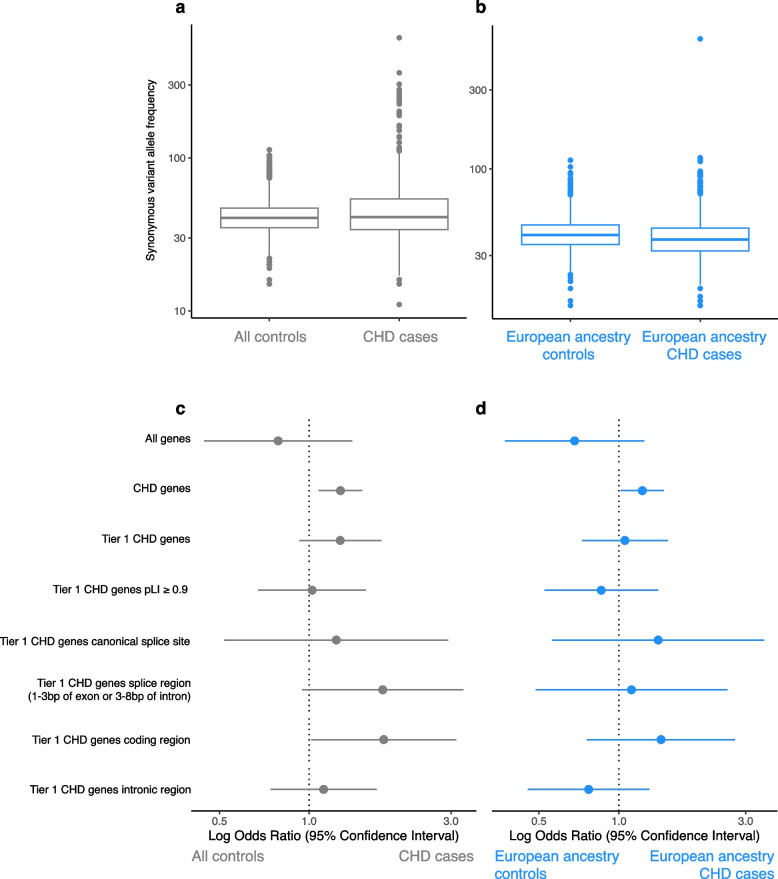
CHD case–control burden of splice-disrupting variants. The burden of variants was compared between 947 CHD cases vs 2570 healthy controls. *Synonymous variants*: The per-sample allele frequency of synonymous variants is shown for **a** all samples and **b** the subset of samples with European genetic ancestry. A median of 41 and 40.5 synonymous variant alleles were found in cases and controls, respectively (*p* > 0.05), indicating that the two cohorts were directly comparable. *Splice-disrupting variants*: High-confidence splice-disrupting variants found in cases and controls were limited to those selected by weighted model 4, and annotated according to the intragenic region they were located at. Odds ratios and 95% confidence intervals are shown comparing variant burden in CHD cases and controls for **c** all samples and **d** the subset of samples with European genetic ancestry. Splice-disrupting variants were predominantly enriched in CHD genes, especially Tier 1 CHD genes. CHD, congenital heart disease; pLI, probability of being loss-of-function intolerant

We compared the characteristics of splice-disrupting variants in CHD patients to controls without CHD utilizing case–control burden analyses. We first assessed the burden of rare, genome-wide, autosomal, synonymous protein-coding variants in 947 *Extension* cohort CHD cases vs 2570 healthy controls with GS. This analysis was performed to ensure that differences in non-synonymous variant burden were not secondary to technical sequencing and workflow differences. Additionally, a stringent gnomAD PopMax threshold was used to ensure that differences in cohort ancestries did not drive differences in burden between cohorts. Moreover, both gnomAD v2 and v3 thresholds were used in order to account for differences in variant frequencies of reference genomes between the *Extension* and *Control* cohorts. This analysis confirmed that the two cohorts had a similar burden of synonymous variants (medians of 41 and 40.5 rare, synonymous variant alleles per sample for the *Extension* and *Control* cohorts respectively, nominal *p* > 0.05). We next assessed the burden of rare, splice-disrupting (SpliceAI Δ score ≥ 0.2) high-confidence variants selected by weighted random forest model 4, in the *CHD Extension* versus *Control* cohorts. Genome-wide, we did not observe a significant difference (nominal *p* > 0.05) in variant burden in cases versus controls (Fig. 9). However, there was a significantly higher burden in cases versus controls of splice-disrupting variants in all Tier 1 and 2 CHD genes (nominal *p* = 0.005). On subgroup analysis, cases had a higher frequency of variants located at canonical splice sites, splice regions, protein-coding regions, and intronic regions of Tier 1 CHD genes, although these differences did not reach statistical significance. This may be due in part to the low variant numbers within each of the gene gene sub-regions, resulting in reduced statistical power. While the *Extension* and *Control* cohorts had a similar proportion of male participants (56 and 49%, respectively), there was a lower proportion of samples of European descent in cases compared with controls (78 and 96%, respectively). To eliminate confounding related to ancestral differences between cases and controls, we did subgroup analysis in individuals of European descent and observed similar trends, with a significantly higher burden of high-confidence splice-disrupting variants among CHD genes despite a reduced statistical power from the smaller sample size (nominal *p* = 0.04).

In summary, a heart-specific model identified high-confidence splice-disrupting variants in CHD genes at canonical splice sites in 1% all CHD cases and non-canonical variants in 11% cases, with splice-disrupting variants accounting for up to 75% of all putatively disease-causing variants in CHD cases (Fig. 5). In particular, deeply intronic cryptic splice variants represented 49% of all high-confidence DNA splicing variants in CHD genes.

## Discussion

Our study applied genome sequencing coupled with myocardial RNA sequencing to identify and validate splice-disrupting variants, including non-canonical splice variants, associated with CHD. Splice-disrupting variants in CHD genes were enriched in cases compared to controls and were associated with altered myocardial gene splicing. The findings were leveraged to develop a machine learning model to predict cardiac-specific, high-confidence non-canonical splice variants. Together, this model identified disease-associated splice-disrupting variants in 12% of CHD patients (75% of all putatively pathogenic CHD associated variants), representing a significant expansion of the role of these variants in heart disease.

RNA-Seq of patient myocardium enabled us to directly identify splice-disrupting events across the genomes of TOF probands. By integrating these validated events with DNA variants obtained from GS, we generated an in silico model that predicted heart-specific splice-disrupting variants with greater accuracy than using models based on SpliceAI alone. Importantly, model performance was validated in an independent CHD cohort. Application of the model to an additional 947 CHD GS samples identified 11% of CHD cases that harbored non-canonical splice-disrupting variants in Tier 1 or 2 CHD genes. As nearly half (49%) of these variants were intronic, our combined use of RNA-Seq and GS enabled the identification of putatively pathogenic variants that would not be detected by panel or exome sequencing, as intronic variants beyond ~ 50 bp are not reliably found with these methods [14, 15].

A recent exome sequencing study highlighted a role for splice-disrupting variants in CHD [11]. In particular, 2% of genome-wide, de novo, computationally predicted splice-disrupting variants in CHD probands were validated by minigene assays. In addition, in a case–control burden analysis, an enrichment for rare splice region variants predicted to result in the loss of nearby existing splice junctions among CHD genes was observed. Unfortunately, exome sequencing is unable to detect deeply intronic splice-disrupting variants [14, 15], and minigene assays alone have technical limitations since they are not cardiac specific, cannot test variants in repetitive regions, and often provide indeterminate results [75, 76].

In this regard, our machine learning model was highly accurate for identifying non-canonical variants that result in confirmed splicing events specific to the human heart (AUC = 0.86). When applied to independent cohorts of TOF and cardiomyopathy, the model was able to identify non-canonical variants that affect splicing in CHD and cardiomyopathy genes respectively. This approach allowed us to recover deeply intronic cryptic splicing variants that cannot be captured by exome sequencing, and have not been previously reported. Of note, direct investigation of patient myocardium identified aberrant splicing events in CHD and other cardiac genes even when a causal DNA variant responsible for the effect could not be definitively confirmed. This may be due to the causal variant having a predicted SpliceAI Δ score below our minimum threshold (0.2), the variant affecting splicing at a greater distance than our maximum threshold (100 bp), or somatic mosaicism resulting in variants in the heart that are undetectable in blood and/or saliva [77]. Deciphering the nature of these tissue-only events requires further study.

Seventy nine percent of predicted high-confidence Tier 1 CHD splice-disrupting variants occurred in genes with associations to syndromic disease that had not been clinically identified. Intriguingly, an intronic de novo variant in *CHD7* was observed in a TOF proband who had phenotypic features consistent with CHARGE syndrome which is caused by *CHD7* variants. Yet, the patient had tested negative on previous clinical genetic testing for *CHD7* variants (Fig. 6). The intronic *CHD7* variant found on research GS (coupled with phenotypic concordance with CHARGE syndrome) was adjudicated by our clinical genetics committee to be likely pathogenic, was subsequently confirmed on clinical genetic testing and returned to the family, resulting in the resolution of the diagnostic odyssey for this patient. Crucially, a prediction model trained only on SpliceAI scores failed to select this variant, again reinforcing the value of using a heart-specific prediction model. In other patients, splice-disrupting events were observed in the syndromic genes *EFTUD2* and *MAP2K1* (Fig. 8), with patient phenotypes consistent with known genotype–phenotype associations.

While at this time, most non-canonical splice-disrupting variants must be functionally validated in order to be considered pathogenic/likely pathogenic, our use of variant segregation and deep phenotyping meant that we were able to classify variants like the aforementioned *CHD7 *de novo variant as likely pathogenic. As computational tools continue to improve the accuracy of variant selection with specific effects on splicing, it may allow for more streamlined clinical reporting of such variants. Indeed, our results support and extend on the recently published ACMG/AMP framework for validating and reporting splice-disrupting variants, including those outside of canonical splice sites [13]. In particular, our findings reinforce the utility of using heart-specific models trained on patient myocardium to improve the accuracy of variant selection in CHD. Moreover, our observation that deeply intronic cryptic splice variants contribute to CHD highlight the necessity of including these types of variants in clinical tests. The inclusion of our validated variant selection model into clinical GS workflows has the potential to increase the diagnostic yield for CHD and to reduce the number of variants that would require functional validation, which can be a time-consuming and expensive endeavor, and limited by availability of relevant patient-derived tissue. Simultaneously, our strategy offers to increase sensitivity for selecting splice-disrupting variants that are not in close proximity to previously annotated splice junctions. As many standard panel and exome tests do not capture and report these variants, the shift to clinical GS tests will better identify such deeply intronic splice-disrupting variants, and will in turn provide supporting evidence for how these genes and variants contribute to the genetic etiology of CHD.

Our study had some limitations. (i) While RNA-Seq of patient myocardium allowed us to directly identify altered splicing events in vivo, these types of events may sometimes lead to nonsense-mediated decay, thereby limiting the ability to detect them in patient tissue although only 19% of Tier 1 and 22% of Tier 2 CHD genes had very low expression (TPM < 1). While in vitro methods for the inhibition of nonsense-medicated decay can be used to validate splicing effects, they are not feasible to test for a large number of variants. Similarly, CHD genes and transcriptional isoforms expressed during embryogenesis but not in mature patient myocardium may not have been detectable in our RNA-Seq data which may also have resulted in underestimation of some splice-disrupting variants. (ii) Another limitation is that short-read sequencing for both RNA-Seq and GS may have missed variants in homologous and low complexity regions, due to unreliable alignments in such regions. Similar studies performed with higher depth sequencing and/or long-read sequencing may further extend our ability to reliably detect splice-disrupting events in known and candidate CHD genes. (iii) We were unable to resolve false negative calls which may have occurred as a result of very low SpliceAI scores, altered splicing occurring more than 100 bp from a variant, or trans-acting effects caused by, for example, changes to the spliceosome. (iv) Although all variants in the *Discovery* cohort were subsequently excluded from validation, it is possible that new variants tested by our models had such similar profiles to those used in training so as to constitute “mirroring.” (v) Our study was limited to rare splice-disrupting events and therefore more common events, including shifts between well-annotated RNA splice isoforms, may have been missed. (vi) Finally, we acknowledge technical differences including sequencing facility, GS platform, reference genome version, and variant detection workflows between cases and controls have the potential to confound variant burden testing results. However, there was no difference in the burden of synonymous variants between cases and controls suggesting that the observed differences in the burden of rare variants was not related to technical sequencing differences.

## Conclusions

By coupling myocardial RNA-Seq and GS data, we developed and validated a cardiac-specific machine-learning model that identified high-confidence canonical and non-canonical splice-disrupting variants associated with CHD. The high burden of non-canonical splice-disrupting variants in patients with CHD makes a strong case for the use of GS to facilitate evaluation of non-canonical sites, including deeply intronic regions.

## Supplementary Information

Additional file1: Table S1. Study cohorts. Clinical characteristics of congenital heart disease probands in the CHD *Discovery*, *Validation*, and *Extension* cohorts (*n*=1,101), as well as characteristics of the cardiomyopathy *Validation* (*n*=43) and Medical Genome Reference Bank *Control* cohort (*n*=2,570).

Additional file2: Table S2. CHD gene list. Genes were categorized into Tier 1 (moderate to strong association with CHD) or Tier 2 (limited association with CHD). Gene Median gene expression (Transcripts Per Million) was calculated for the entire *Discovery* cohort. Gene annotations and constraint metrics are additionally shown.

Additional file3: Table S3. Myocardial RNA outlier splicing events and confirmed associated DNA splice variants in CHD *Discovery* cohort (*n*=106). 100 rare (internal MAF < 0.01) genome-wide DNA splice-disrupting variants within the *Discovery* cohort were confirmed by myocardial RNA-Seq. In addition, six significant RNA splicing events were observed without a causative DNA splice-disrupting variant in Tier 1 CHD genes or haploinsufficiency-intolerant (pLI≥0.9) Tier 2 CHD genes. All variant features used in random forest models are included. Clinical features of the proband harboring each RNA splicing event are additionally shown.

Additional file4: Table S4. Performance of random forest models using five-fold cross-validation in CHD *Discovery* cohort (*n*=106). Contingency tables and associated statistics are shown for whether variants were selected by random forest models and whether splicing was confirmed by FRASER.

Additional file5: Table S5. High-confidence DNA splicing variants within the CHD (*n*=48) and cardiomyopathy (*n*=43) *Validation* cohorts. All genome-wide high-confidence DNA variants in the CHD and cardiomyopathy *Validation* cohorts selected by weighted and SMOTE random forest model 4 are shown, along with confirmed variants that were not selected by the model. Variants are annotated by their matching splicing and gene expression outlier statistics obtained from FRASER and OUTRIDER, respectively.

Additional file6: Table S6. Performance of random forest models in CHD (*n*=48) and cardiomyopathy (*n*=43) *Validation* cohorts. Contingency tables and associated statistics are shown for whether variants were selected by random forest models and whether splicing was confirmed by FRASER.

Additional file7: Table S7. High-confidence DNA splice variants in CHD genes in CHD *Extension* cohort (*n*=947). Rare (gnomAD v2 allele frequency < 0.0001 and gnomAD v3 PopMax allele frequency < 0.0001) high-confidence splice-disrupting DNA variants in Tier 1 CHD genes or haploinsufficiency-intolerant (pLI≥0.9) Tier 2 CHD genes were identified in the *Extension* cohort. DNA variants were selected by weighted random forest model 4 (*Extension* cohort), yielding an additional 42 variants in Tier 1 CHD genes and 79 variants in Tier 2 CHD genes. All variant features used in random forest models are included. Clinical features of the proband harboring each DNA variant are additionally shown.

Additional file8: Table S8. Gene sets enriched for genome-wide high-confidence splicing variants in CHD genes in the CHD *Discovery*, *Validation*, and *Extension* cohorts (*n*=1,101). 133 high-confidence splice-disrupting variants in CHD genes were identified in the 1,101 CHD patients - *Discovery* (*n*=106), *Validation* (*n*=48), and *Extension* (*n*=947) cohorts. Variants were tested for enrichment within Human Phenotype Ontology gene sets. Significantly enriched terms (adjusted *p* < 0.01) are shown.

## Data Availability

Variant data for the *Discovery*, *CHD Validation*, and *Extension* cohorts from all three biobank registries is available in the European Genome-Phenome Archive (EGA) under accession EGAS50000000586 (https://ega-archive.org/studies/EGAS50000000586) [16], and will be available for download upon approval by the Data Access Committee (https://theheartcentrebiobank.com/sample-request). Sequencing data for the cardiomyopathy *Validation* cohort is available in EGA under accession EGAS00001004929 (https://ega-archive.org/studies/EGAS00001004929) [6], and are available for download upon approval by the Data Access Committee (https://theheartcentrebiobank.com/sample-request). Sample level Variant Call Format (VCF) files for Canadian and Australian cohorts are available on the seqr platform [78] and can be accessed at https://seqr.ccm.sickkids.ca. For sample-level genomic data from the Netherlands cohort, request should be sent to the PI (C.B.) due to consent restrictions. *Control* cohort MGRB data are available by controlled access in EGA under accession EGAS00001003511 (https://ega-archive.org/studies/EGAS00001003511) [17]. Computational workflow code is available on GitHub (https://github.com/mitallab/CHD_Splicing_Lesurf). Additional data generated or analyzed during this study are included in the supplementary information files, and additional raw data used for figures and results are available from the corresponding author on reasonable request.

## Abbreviations

CHD: Congenital heart disease
TOF: Tetralogy of Fallot
TGA: Dextro-transposition of the great arteries
ACMG/AMP: American College of Medical Genetics and Genomics and the Association for Molecular Pathology
GS: Genome sequencing
RNA-Seq: RNA-sequencing
MGRB: Medical Genome Reference Bank
SNV: Single-nucleotide variant
indel: Insertion-deletion
TCAG: The Centre for Applied Genomics
GATK: Genome Analysis Toolkit
gVCF: Genotype Variant Call Format
VQSR: Variant quality score recalibration
GQ: Genotype Quality
CNV: Copy number variant
SV: Structural variant
OMIM: Online Mendelian Inheritance in Man
ClinGen: Clinical Genome Resource
MANE: Matched Annotation from NCBI and EMBL-EBI
HGMD: Human Gene Mutation Database
DFP: Disease-associated polymorphism with supporting functional evidence
DP: Disease-associated polymorphism
DM: Disease causing mutation
o/e: Observed/expected
LoF: Loss-of-function
gnomAD: Genome Aggregation Database
IGV: Integrative Genomics Viewer
CMP: Cardiomyopathy
AUC: Area under the curve
SCS: Set Counts and Sizes
MAF: Minor allele frequency
TPM: Transcript per million
